# The Effect of Ramadan and COVID-19 on the Relationship between Physical Activity and Burnout among Teachers

**DOI:** 10.3390/nu14132648

**Published:** 2022-06-26

**Authors:** Maamer Slimani, Hela Znazen, Fairouz Azaiez, Nicola Luigi Bragazzi

**Affiliations:** 1 Postgraduate School of Public Health, Department of Health Sciences (DISSAL), Genoa University, 16132 Genoa, Italy; robertobragazzi@gmail.com; 2 Department of Physical Education and Sport, College of Education, Taif University, Taif 21944, Saudi Arabia; znazen.hela@laposte.net; 3 Group for the Study of Development and Social Environment (GEDES), Faculty of Human and Social Science of Tunis, University Tunis, Tunis 1007, Tunisia; fairouz.kyranis@yahoo.com; 4 Department of Human Sciences, Higher Institute of Sport and Physical Education of Sfax, University of Sfax, Sfax 3000, Tunisia; 5 Laboratory for Industrial and Applied Mathematics (LIAM), Department of Mathematics and Statistics, York University, Toronto, ON M3J 1P3, Canada

**Keywords:** COVID-19, Ramadan, education, physical activity, burnout

## Abstract

The objective of this study was to explore the effect of COVID-19 and Ramadan on physical activity (PA) and burnout in teachers and the relationship between them. A total of 57 secondary school teachers from public education centers participated in the present study. They were aged between 29 and 52 years. To determine the effect of Ramadan and COVID-19 on PA and burnout, participants completed the online questionnaires before COVID-19, one week before Ramadan and during the second week of Ramadan. The International Physical Activity Questionnaire-BREF and the Maslach Burnout Inventory-Human Services Survey were used to assess PA intensities and burnout, respectively. The data revealed that total PA (*p* < 0.001, *p* < 0.05, respectively) vigorous metabolic equivalent of task (MET) (*p* < 0.001, *p* < 0.05, respectively), moderate MET (*p* < 0.001, *p* < 0.01, respectively) were higher before COVID-19 and before Ramadan than during Ramadan. Regarding burnout subscales, emotional exhaustion (*p* < 0.001, *p* < 0.01, respectively) was higher before Ramadan than before COVID-19 and during Ramadan. A lower personal accomplishment was reported before Ramadan than before COVID-19 and during Ramadan (both *p* < 0.05). In addition, low to high correlations were observed between PA intensities and burnout subscales, except for the correlation between depersonalization and all PA intensities. In conclusion, Ramadan intermittent fasting along with PA was highly recommended for teachers and the general population to improve positive emotions and general health.

## 1. Introduction

Since late 2019–early 2020, the world has been facing an unprecedented situation caused by an emerging coronavirus known as Severe Acute Respiratory Syndrome-related Coronavirus type 2 (SARS-CoV-2), responsible for the Coronavirus Disease 2019 (COVID-19) outbreak, which has spread worldwide, sparing virtually no country or territory. Its quick and increasing transmissibility has made the World Health Organization (WHO) recommend the adoption and implementation of some non-pharmaceutical interventions (NPIs), including physical or social distancing, self-isolation, stay-at-home orders and quarantine, and the ban and cancellation or postponement of sporting events and education, which have gone online in the effort to curb the diffusion of COVID-19 [1]. These interventions have had, on the one hand, a positive effect in terms of reduced disease transmission, but, on the other hand, have exerted a negative impact on many sectors, such as the economy and teaching. In the latter context, NPIs may have dual effects in that they may decrease the transmission of the virus between teachers and students. In a negative way, teachers have experienced a new working format and lifestyle behaviors. This challenge may force teachers to use digital technologies, spend more time sitting, modify or increase their workload, and become inactive. These aspects can have a significant impact on psychological health and may increase the risk of diseases, such as obesity and metabolic impairment, among teachers.

Psychological health also refers to the opportunities that the organizational context offers to the individual, the working conditions and tasks assigned to them, to feel valued, to develop, to lead a balanced life, and to have a level of stress that they are able to manage [2]. For Foucher and Leduc [3], it is the state that an individual develops in relation to the indices of self-esteem or valorization, fulfillment, the balance of life, and stress level during different life situations. Morin [4] points out that mental health is not only the absence of mental disorders, but is also a state of well-being in which each person realizes their potential, faces the normal difficulties of life, works productively, and can contribute to the community. Although there is not always a clear and integrated conceptualization of this construct [5], psychological health is now understood as a multidimensional construct that reflects both the absence of negative manifestations and the presence of positive manifestations [6,7].

In this context, some studies dealing with the COVID-19 pandemic reported that this situation has a negative effect on the psychological health of both students and teachers [8]. A study carried out in an Arabic country showed that this pandemic has negative effects on teachers in terms of increased rates of depression, anxiety, and divorce, all of which limit their ability to teach in a proper way [9]. Another investigation conducted in China reported that the rate of stress symptoms was 9.1% among teachers, which underlines the importance of adopting an intervention that supports them psychologically [10]. Moreover, the changes in psychological states were affected by many factors such as food restriction, sleep loss, and physical inactivity during COVID-19 [11].

The Islamic month of Ramadan is characterized by intermittent fasting, with changes in eating behavior, sleep, and physical activity (PA) patterns. Many studies reported changes in energy balance [12] and physical performance [13]. However, few studies have quantitatively assessed the effect of Ramadan on psychological health/states [14,15,16]. For instance, there is no study that investigated the effect of intermittent fasting/Ramadan and PA on psychological states during COVID-19 and their relationship. Therefore, the objective of the present investigation was to explore the effect of COVID-19 and Ramadan on PA and burnout in teachers and the relationship between them.

## 2. Materials and Methods

### 2.1. Participants

A total of 57 secondary school teachers from public education participated in the present study. They were aged between 29 and 52 years. Most of the participants were males (80.7%) and married (19.3%). In terms of specialty, 40.3% were teaching scientific topics, and 59.7% teaching physical education. In terms of sector, all of them were teaching in Secondary Education. All participants were extensively advised about the aim of the present investigation. This study received ethical clearance by the Ethics Committee of Taif University, Saudi Arabia, and by the UNESCO Chair “Health Anthropology Biosphere and Healing Systems,” University of Genoa, Genoa, Italy (project code EXERCOGN_023020), and it was carried out according to the 1964 Declaration of Helsinki and its subsequent amendments.

To determine the effect of Ramadan, which took place between 13 April and 12 May 2021, and COVID-19 on PA and burnout, participants completed the online questionnaires during the second week of Ramadan in comparison with before COVID-19, and one week before Ramadan, as suggested by previous studies [17,18].

### 2.2. Measures

#### 2.2.1. Physical Activity

The International Physical Activity Questionnaire-BREF (IPAQ-BREF [19]) was used to assess PA. The questionnaire consisted of 7 items: 6 of them assessed the duration (in minutes) and frequency (from 1 to 7 days) of vigorous-intensity, moderate-intensity, and walking PA for at least 10 min during the past week. The last question was “During the last 7 days, how much time did you spend sitting on a week day?”. Of note, the intensity of activity was converted to the metabolic equivalent of task (MET) units (MET^−h^·week^−1^), following the recommendations of a previous study [20].

#### 2.2.2. Burnout

The Maslach Burnout Inventory-Human Services Survey (MBI-HSS) questionnaire has been widely used to assess burnout among the English population [21]. In this study, we used the validated version in the Arabic language [22,23,24]. This questionnaire contains 22 items grouped into three scales: emotional exhaustion (9 items), depersonalization (5 items), and (reduced) personal accomplishment (8 items). The items are graded on a 7-point Likert scale ranging from 0 (never) to 6 (daily) based on how often one experiences these feelings. The Cronbach’s alpha coefficient (α) that tested the internal consistency of the three subscales ranged between 0.74 and 0.90, as obtained in the current and previous studies [22,25].

### 2.3. Statistical Analysis

Descriptive statistical analysis was conducted by computing the means and standard deviations for each of the parameters under study. One-way analysis of variance (ANOVA) and Pearson’s correlation coefficient were used to capture differences between before and after confinement and the correlation between variables. The strength of the correlation was considered negligible (*r* coefficient ranging from 0.00 to 0.29), low (*r* ranging from 0.30 to 0.49), moderate (r ranging from 0.50 to 0.69), high (r ranging from 0.70 to 0.89), and very high (*r* ranging from 0.90 to 1.00) [26]. All statistical analyses were carried out using the commercial software “Statistical Package for Social Sciences” (SPSS version 24.0, IBM, Armonk, NY, USA). Results with *p*-values less than or equal to the threshold of 0.05 were considered statistically significant.

## 3. Results

One-way ANOVA reported a significant effect between groups in total PA (F = 8.15, *p* < 0.001), vigorous MET (F = 10.56, *p* < 0.001), and moderate MET (F = 8.56, *p* < 0.001). Post-hoc analysis revealed that total PA (*p* < 0.001, *p* < 0.05, respectively) vigorous MET (*p* < 0.001, *p* < 0.05, respectively), moderate MET (*p* < 0.001, *p* < 0.01, respectively) were higher before COVID-19 and before Ramadan than during Ramadan. No statistically significant difference between before COVID-19 and before Ramadan in all of the above-mentioned parameters (all, *p* > 0.05) could be found (Table 1).

Regarding burnout subscales, a significant difference between groups was reported in emotional exhaustion (F = 10.87, *p* < 0.001), and personal accomplishment (F = 4.98, *p* < 0.01). Post-hoc analysis revealed that emotional exhaustion was higher before Ramadan than before COVID-19 and during Ramadan (*p* < 0.001, and *p* < 0.01, respectively). A lower personal accomplishment was reported before Ramadan than before COVID-19 and during Ramadan (both *p* < 0.05). No significant difference between before COVID-19 and during Ramadan in both subscales (all *p* > 0.05) could be detected (Table 1).

Furthermore, no statistically significant difference between groups regarding walking MET (F = 2.38, *p* = 0.096) and depersonalization (F = 0.28, *p* = 0.75) was described.

Before COVID-19, the results showed significant correlations between total PA and burnout subscales. Low to high correlations (-0.35 to 0.87) were observed between PA intensities and burnout subscales, except for the correlation between depersonalization and all PA intensities (*p* > 0.05).

Similarly, during COVID-19, the results showed significant correlations between total PA and burnout subscales. Low to high correlations were observed between PA intensities and burnout subscales, except for the correlation between depersonalization and all PA intensities (Table 2).

During Ramadan, the results showed significant correlations between total PA and burnout subscales: specifically, the correlation was negative with emotional exhaustion (*r* = −0.61, *p* < 0.001) and positive with personal accomplishment (*r* = 0.51, *p* < 0.001) (Table 3).

## 4. Discussion

The present investigation aimed to explore the effect of COVID-19 on burnout and physical activity during Ramadan 2021 in teachers. The diffusion of COVID-19 has a detrimental effect on the psychological states, specifically burnout, of teachers. A study done in Morocco showed that 54% of teachers experienced low to severe levels of burnout in response to COVID-19 [25]. From the start of the pandemic, many researchers analyzed the psychological consequences. A first study carried out in China in January 2020 assessed the severity of psychological distress felt during COVID-19-induced confinement [27]. This study assessed the frequency of anxiety, depression, avoidance behaviors, and physical symptoms onset at the time of the last week of testing. 

The results showed a moderate level of psychological distress for 35% of the population, while 5% had a high level [27]. A systematic review of the literature on the psychological impact of quarantine, published on 14 March 2020 in the Lancet [8], screened 3166 articles, retaining 24 studies with scientific solidity from ten countries and investigating SARS, Ebola, swine flu (H1N1), and equine influenza. The authors highlighted the existence of two types of stress factors, those linked to confinement itself and those linked to the consequences of confinement. No confinement period in these studies was longer than 3 weeks. Analysis of the results of the studies showed a high prevalence of symptoms of distress, depression, stress, irritability, anxiety-type insomnia, anger, emotional exhaustion, symptoms of post-traumatic stress, fear, loss, and culpability. Some symptoms persisted for several months after the lifting of the confinement, reflecting a continuation in behaviors of avoidance of physical contact, due to fear of crowd gatherings in public spaces and of taking public transport. In people with mental fragility before the quarantine, maintenance of anxiety and anger 4 to 6 months after the end of the quarantine could be observed. Healthcare professionals showed much more severe post-traumatic stress symptoms than the general population and were much more morally affected. Three studies out of the 24 analyzed made a link between the duration of quarantine and after effects, showing that a duration longer than 10 days was more likely to cause higher post-traumatic stress levels than a shorter period.

When comparing burnout subscales during COVID-19 before Ramadan and during Ramadan, emotional exhaustion was higher during COVID-19 than during Ramadan, while the opposite was found for personal accomplishment. Confirming the data of the present study, some authors reported that Ramadan fasting could decrease depression, anxiety, and stress scores and increase mood and vitality [16,28,29,30,31].

By discriminating between different intensities of PA, this study demonstrates that total, vigorous, and moderate MET were higher before and during COVID-19 than during Ramadan. In addition, they correlated with emotional exhaustion and personal accomplishment, which may be considered a buffering effect against teacher burnout (i.e., emotional exhaustion) [32]. This investigation is in accordance, in part, with a previous study, which reported that low, medium, and high PA negatively correlated with total burnout score and subscale of exhaustion [33]. These findings are of paramount importance because being regularly engaged in PA has been found to be a powerful approach for alleviating occupational stress in general [34,35] and teacher stress specifically [36,37,38]. Thus, it appears that PA participation is not something that you have to wait until after work; you can and should do it during work. These findings corroborate the crucial role of PA in preventing and mitigating negative stress responses, thereby confirming Salmon’s [39] unifying theory concerning childcare teachers. In the same way, the role of PA in the general health and well-being of teachers during lockdown should be important also, as it has been found that, in those teachers who perform more exercise during leisure time, or more autonomously, it may prevent physical and mental health problems [40].

## 5. Conclusions

In conclusion, this is the first study to show that COVID-19 has a negative effect on burnout subscales (i.e., emotional exhaustion and personal accomplishment) among teachers. In addition, despite the fact that Ramadan may decrease physical activities (i.e., vigorous and moderate MET) among teachers and their significant correlations with emotional exhaustion and personal accomplishment, it has been shown that Ramadan has a positive effect on burnout. Ramadan intermittent fasting, along with PA, was highly recommended for teachers and the general population to improve positive emotions and general health.

## Figures and Tables

**Table 1:** Values of physical activity and burnout subscales, broken down according to the timing.

Variable	Before COVID-19	Before Ramadan	During Ramadan	Statistical Significance
Vigorous MET	312.98 ± 442.01	213.33 ± 256.49	49.12 ± 161.95	*p* < 0.001
Moderate MET	192.98 ± 238.34	168.07 ± 238.68	47.01 ± 89.16	*p* < 0.001
Walking MET	680.26 ± 823.27	536.68 ± 640.48	393.97 ± 619.30	*p* = 0.096
Total PA	1186.22 ± 1130.68	918.08 ± 938.60	490.11 ± 652.29	*p* < 0.001
Emotional exhaustion	9.01 ± 6.81	11.03 ± 6.76	14.85 ± 6.79	*p* < 0.001
Personal accomplishment	22.82 ± 7.77	19.14 ± 6.52	22.73 ± 6.97	*p* = 0.008
Depersonalization	2.59 ± 3.16	3.12 ± 3.84	2.70 ± 3.90	*p* = 0.75

MET, metabolic equivalent of task; PA, physical activity.

**Table 2:** Correlations between physical activity (PA) levels and burnout subscales among participants during COVID-19 or before Ramadan.

	Total PA	Vigorous MET	Moderate MET	Walking MET	Emotional Exhaustion	Depersonalization	Personal Accomplishment
**Total PA**	1						
**Vigorous MET**	0.51 (<0.001)	1					
**Moderate MET**	0.59 (<0.001)	0.23 (0.05)	1				
**Walking MET**	0.64 (<0.001)	0.15 (0.2)	0.31 (0.03)	1			
**Emotional exhaustion**	−0.58 (<0.001)	−0.34 (0.004)	−0.78 (<0.001)	−0.57 (<0.001)	1		
**Depersonalization**	−0.21 (0.07)	−0.23 (0.26)	−0.20 (0.06)	−0.19 (0.09)	0.35 (<0.001)	1	
**Personal accomplishment**	0.61 (<0.001)	0.37 (<0.001)	0.84 (<0.001)	0.65 (<0.001)	−0.68 (<0.001)	−0.41 (<0.001)	1

MET, metabolic equivalent of task; PA, physical activity.

**Table 3:** Correlations between physical activity (PA) levels and burnout subscales among participants during Ramadan.

	Total PA	Vigorous MET	Moderate MET	Walking MET	Emotional Exhaustion	Depersonalization	Personal Accomplishment
**Total PA**	1						
**Vigorous MET**	0.66 (<0.001)	1					
**Moderate MET**	0.67 (<0.001)	0.41 (<0.001)	1				
**Walking MET**	0.72 (<0.001)	0.13 (0.3)	0.20 (0.13)	1			
**Emotional exhaustion**	−0.47 (<0.001)	−0.32 (0.01)	−0.72 (<0.001)	−0.45 (<0.001)	1		
**Depersonalization**	−0.24 (0.06)	−0.19 (0.12)	−0.25 (0.056)	−0.23 (0.08)	0.32 (0.002)	1	
**Personal accomplishment**	0.51 (<0.001)	0.32 (0.01)	0.76 (<0.001)	0.54 (<0.001)	−0.61 (<0.001)	−0.45 (<0.001)	1

MET, metabolic equivalent of task; PA, physical activity.

## Data Availability

The data are not publicly available due to privacy or ethical reasons.
